# Clinical Outcomes of Single-Port Versus Conventional Multiport Laparoscopic Myomectomy: A Retrospective Study

**DOI:** 10.1177/26884844251383823

**Published:** 2025-09-30

**Authors:** Yucui Zeng, Ping Li, Yushan Li, Wenwei Luo, Xiaoyan Guang

**Affiliations:** Department of Obstetrics and Gynecology, Peking University Shenzhen Hospital, Shenzhen, China.

**Keywords:** single-port laparoscopy, multiport laparoscopy, myomectomy, minimally invasive surgical technique, uterine fibroids, gynecological surgery

## Abstract

**Introduction:**

To compare the clinical outcomes of laparoscopic myomectomy *via* single-port approach (SPLM) and conventional multiport laparoscopic myomectomy (MPLM).

**Methods:**

A retrospective analysis was conducted on 149 patients with uterine fibroids, including 77 who underwent MPLM and 72 who underwent SPLM. Patient baseline characteristics and perioperative indicators were compared to evaluate surgical efficacy.

**Results:**

No significant differences were observed between the two groups in demographic characteristics, fibroid number, or maximum fibroid diameter (*p* > 0.05). The SPLM group had a higher proportion of anterior wall fibroids (45.8% vs. 26.0%) and subserosal fibroids (38.9% vs. 18.2%) compared to the MPLM group (*p* < 0.05). The SPLM group showed significantly better outcomes in total operative time (99.85 ± 33.65 minutes vs. 120.91 ± 49.48 minutes), time to postoperative gastrointestinal recovery (1.40 ± 0.55 days vs. 1.69 ± 0.47 days), and 24-hour postoperative visual analog scale (VAS) score (2.33 ± 0.65 vs. 2.70 ± 0.84) (*p* < 0.05). No significant differences were found in intraoperative blood loss, postoperative complication rates, or hospital stay between the two groups in the overall analysis. However, based on the four influencing factors, further stratified analysis within each factor revealed that SPLM was more advantageous for treating single fibroids, fibroids less than 8 cm in diameter, and intramural fibroids, demonstrating significantly shorter operative times than MPLM (*p* < 0.05). Surgeon A demonstrated superior outcomes in both operative time and intraoperative blood loss when performing myomectomy *via* the single-port laparoscopic approach compared to the multiport technique (*p* ≤ 0.05).

**Conclusion:**

SPLM is a safe and effective surgical approach. For single intramural fibroids less than 8 cm in diameter, it demonstrates superior operative efficiency compared to conventional MPLM, particularly regarding operative time. When performed by the senior surgeon, the advantages of SPLM become even more pronounced in both operative duration and intraoperative blood loss.

## Introduction

Uterine fibroids, the most common benign uterine tumors in women, exhibit significant regional variations in prevalence among reproductive-aged women, with reported incidence rates ranging from 5.4% to 77%.^1^ These tumors may lead to multiple complications such as menorrhagia, prolonged menstrual bleeding, dysmenorrhea, pelvic pressure symptoms, and infertility.^2^ Clinically, multiple treatment options exist for uterine fibroids, with myomectomy remaining the preferred surgical approach for women of reproductive age. Since its initial clinical application in 1979,^3^ laparoscopic technology has undergone refinement for decades. Guided by modern enhanced recovery protocols,^4^ laparoscopic myomectomy has become a well-established technique for managing benign gynecological diseases.

Laparoscopic myomectomy offers effective treatment while eliminating the large abdominal incisions associated with traditional open surgery. In conventional procedures, fibroid specimens are typically extracted through an enlarged trocar site in the left lower abdomen using a laparoscopic power morcellator. However, following the Food and Drug Administration (FDA) explicit warning against power morcellator use,^5^ gynecologists have explored alternative minimally invasive approaches. This has led to the development of single-port laparoscopic myomectomy (SPLM). It should be noted that this technique presents certain technical challenges, including limited operative space, instrument crowding, commonly known as the ‘chopstick effect’, and a steep learning curve,^6^ which has made many gynecologists hesitant to adopt it. Nevertheless, substantial clinical evidence has demonstrated the safety and efficacy of SPLM.^7–9^ With its dual advantages of minimally invasive properties and superior cosmetic results, the technique is gaining increasing clinical acceptance.

Our center has successfully implemented single-port laparoscopic procedures in gynecology and developed considerable expertise in this technique. In this study, we compare perioperative outcomes between patients undergoing SPLM and conventional multiport laparoscopic myomectomy (MPLM) at our department over the past 2 years. This comparison further demonstrates the clinical value of SPLM.

## Methods

### Patients

From August 2022 to July 2024, 72 patients underwent SPLM at the Department of Gynecology, Peking University Shenzhen Hospital, while a control group of 77 patients received MPLM. All surgeries were performed by three senior specialists in gynecological minimally invasive surgery. The research team analyzed relevant clinical indicators by reviewing medical records and surgical videos. This study was approved by the Ethics Committee of Peking University Shenzhen Hospital, adhering to the Declaration of Helsinki. Informed consent was obtained from all subjects before the surgery.

All patients underwent preoperative gynecological three-dimensional ultrasound examinations to assess uterine fibroid size, location, and classification based on the International Federation of Gynecology and Obstetrics (FIGO) fibroid classification.^10^ Both groups had well-defined indications for laparoscopic surgery, including menorrhagia, prolonged menstruation, pressure-related symptoms (such as urinary frequency or pelvic discomfort), or infertility. Exclusion criteria included suspected malignancy, inability to tolerate general anesthesia, contraindications to laparoscopic surgery, or cases operated by junior surgeons under the supervision of senior experts.

### Surgical procedure

#### SPLM

**FIG. 1.**
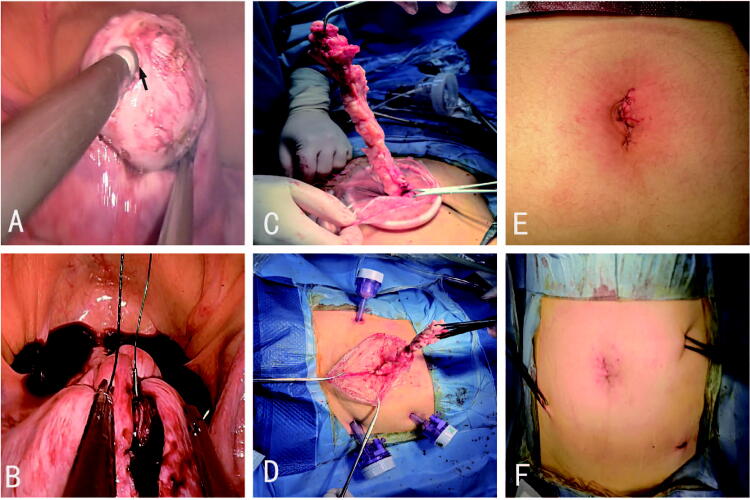
Partial procedure of SPLM and MPLM. **(A)** Blunt dissection of the myoma using a myoma retractor (black arrow). **(B)** Uterine reconstruction using barbed suture with a baseball suture technique. **(C)** and **(D)** The myoma is sharply excised and extracted through the umbilical port. **(E)** Umbilical port closure following SPLM. **(F)** Closure of umbilical and lower abdominal incisions following MPLM. MPLM, multiport laparoscopic myomectomy; SPLM, single-port laparoscopic myomectomy.

All patients were placed in the lithotomy position under general anesthesia. After skin disinfection, a 3 cm vertical incision was made at the umbilicus, and the abdominal cavity was accessed layer by layer. A single-port access device (Kangji, Hangzhou, China) was then inserted, followed by the establishment of a CO_2_ pneumoperitoneum with an intra-abdominal pressure maintained at 12 mmHg. A rigid 30° laparoscope was introduced for a comprehensive exploration of the pelvic and abdominal cavities. After identifying the location of the uterine fibroid, diluted vasopressin (6 IU + 50 mL normal saline) was injected into the adjacent myometrium. Once the medication took effect, the serosal layer of the uterus was incised using monopolar electrocautery (Kangji, Hangzhou, China) until the fibroid capsule was reached. The fibroid was then grasped and initially separated with forceps (Kangji, Hangzhou, China), followed by blunt and sharp dissection using a myoma screw (Kangji, Hangzhou, China) (Fig. 1A). Complete enucleation of the fibroid was achieved. Larger blood vessels encountered during dissection were coagulated using bipolar electrocautery (Kangji, Hangzhou, China) for hemostasis. Careful dissection was performed at the deepest part of the fibroid to avoid penetrating the uterine cavity and causing endometrial injury.

After complete removal of the fibroid, the defect was closed using either full-thickness or layered sutures with barbed absorbable suture (Covidien, Shanghai, China). A baseball suture technique (continuous running suture with alternating directions) was used (Fig. 1B), effectively reshaping the uterus while minimizing barb exposure to prevent bowel adhesion and injury. A specimen retrieval bag was introduced through the umbilical port, and the fibroid was placed inside before being morcellated and extracted *via* the umbilical incision (Fig. 1C). The procedure concluded with thorough irrigation of the pelvic and abdominal cavities and layered closure of the umbilical incision (Fig. 1E).

#### MPLM

In MPLM, a 1.0 cm vertical umbilical incision was made for the placement of a rigid 30° laparoscope. A 1.0 cm main operating port was established in the left lower abdomen, with an additional 0.5 cm auxiliary port in the left upper abdomen. For cases involving large fibroids, an extra 0.5 cm auxiliary port was added in the right lower abdomen. The fibroid removal and suturing techniques mirrored those described for the SPLM above. After enlarging the umbilical incision to 1.5 cm, the fibroid was placed in a specimen bag and morcellated through the enlarged incision. Closure of the umbilical incision was identical with SPLM (Fig. 1D,F).

### Perioperative data

Clinical records were reviewed to collect baseline characteristics and fibroid features, intraoperative data, and postoperative parameters. Operative time was defined as the interval from anesthesia induction to skin closure. Hemoglobin change was calculated by comparing levels measured one day before surgery with those on postoperative day one. Intraoperative blood loss was measured based on the total blood volume in the suction bottle. Postoperative pain was assessed using the VAS for incision pain at 24 hours post-surgery.

### Subgroup analysis

Subgroup analyses were conducted based on four influencing factors: number of fibroids, fibroid diameter, fibroid type, and surgeon. Within each factor, stratified analyses were performed to compare the differences between single-port and multiport laparoscopic surgery in terms of operative time and intraoperative blood loss.

### Statistical analysis

Measurement data are presented as mean ± standard deviation or median (interquartile range, IQR). Independent sample *t*-tests were used for normally distributed measurement data between groups, while the Mann–Whitney U test was applied for non-normally distributed data. Categorical variables were expressed as percentages and analyzed using either the Chi-square test or Fisher’s exact test. A p-value <0.05 was considered statistically significant. All data were analyzed using IBM SPSS Statistics 25.0.

## Results

This study included 149 patients who underwent laparoscopic myomectomy, with 72 in the SPLM group and 77 in the MPLM group. All procedures were successfully completed without conversion to laparotomy.

### Patient baseline characteristics and fibroid features

Patient baseline characteristics are presented in Table 1. No statistically significant differences were observed between the two groups regarding age, BMI, parity, previous pelvic surgery, number of fibroids, or maximum fibroid diameter. Subgroup analysis of fibroid location and classification similarly revealed no significant differences in fibroid distribution between the groups.

**Table 1.** Patient Baseline Characteristics and Fibroid Features of MPLM and SPLM Groups

	Classification	MPLM (*n* = 77)	SPLM (*n* = 72)	*p* value
Age (years)^a^	/	39.31 ± 7.68	37.06 ± 7.01	0.064
BMI (kg/m^2^)^a^	/	22.31 ± 2.56	21.92 ± 2.94	0.388
Parity^b^	Nulliparous	26 (33.8%)	29 (40.3%)	0.410
	Parous	51 (66.2%)	43 (59.7%)	
History of pelvic surgery^b^		29 (37.7%)	19 (26.4%)	0.141
Fibroid features				
Number of fibroids (*n*)^a^	/	1.90 ± 1.12	1.76 ± 1.18	0.484
Size of the largest fibroid (cm)^a^	/	7.47 ± 1.81	7.06 ± 1.68	0.160
Fibroid number^b^	1	40 (51.9%)	44 (61.1%)	0.260
	2	15 (19.5%)	13 (18.1%)	0.824
	3	14 (18.2%)	6 (8.3%)	0.078
	>=4	8 (10.4%)	9 (12.5%)	0.686
Location of largest fibroid^b^	Anterior wall	20 (26%)	33 (45.8%)	0.011
	Posterior wall	30 (39.0%)	24 (33.3%)	0.475
	Lateral wall	11 (14.3%)	5 (6.9%)	0.148
	Uterine fundus	3 (3.9%)	6 (8.3%)	0.315
	Others (broad ligament or cervix)	13 (16.9%)	4 (5.6%)	0.030
Type of largest fibroid^b^	Type 2	1 (1.3%)	0 (0)	0.967
	Type 3	2 (2.8%)	0 (0)	0.497
	Type 4	5 (6.5%)	0 (0)	0.059
	Type 2–5	1 (1.3%)	4 (5.6%)	0.198
	Type 5	38 (49.4%)	23 (31.9%)	0.031
	Type 6	14 (18.2%)	28 (38.9%)	0.005
	Type 7	3 (3.9%)	12 (16.7%)	0.010
	Type 8	12 (15.6%)	5 (6.9%)	0.097
Number of the largest fibroid^b^	<8 cm	38 (49.4%)	45 (62.5%)	0.106
	>=8	39 (50.6%)	27 (37.5%)	

^a^ Data are shown as the mean ± standard deviation.

^b^ *n* (%).

BMI, body mass index; MPLM, multiport laparoscopic myomectomy; SPLM, single-port laparoscopic myomectomy.

Both groups predominantly comprised patients with single fibroids, accounting for 51.9% in the MPLM group and 61.1% in the SPLM group. The anterior and posterior uterine walls represented the most common fibroid locations, comprising over 60% of cases in both groups. Notably, the SPLM group showed a significantly higher proportion of anterior wall fibroids (45.8%) compared to the MPLM group (26.0%), potentially indicating different case selection patterns between the approaches. For fibroids in challenging locations (broad ligament or cervix), the MPLM group demonstrated a significantly higher proportion (16.9%). This may reflect clinicians’ preference for multiport laparoscopy when managing high-risk locations to minimize bleeding risks and avoid conversion.

Regarding fibroid classification, multiport laparoscopy was used for a wider range of types (2–8), while single-port laparoscopy was less frequently employed for types 2–4 due to greater technical challenges. Both groups primarily involved type 5 and 6 fibroids, with type 5 lesions significantly more prevalent in the MPLM group (49.4%) versus the SPLM group (31.9%). Additionally, type 6 fibroids were more common in the SPLM group (38.9%) compared to the MPLM group (18.2%), and type 7 fibroids accounted for 16.7% in the SPLM group, significantly exceeding the 3.9% rate in the MPLM group.

### Perioperative and postoperative outcomes

As shown in Table 2, there was no significant difference in intraoperative blood loss between the two groups (MPLM group: median 50 mL; SPLM group: median 30 mL). However, the SPLM group showed significantly shorter operative time compared to the MPLM group (99.85 ± 33.65 minutes vs. 120.91 ± 49.18 minutes, *p* < 0.05). In the MPLM group, two transfusion cases occurred: one with 1000 mL intraoperative blood loss, and another with preoperative Hb of 80 g/L plus 200 mL intraoperative blood loss. Although one uterine cavity injury occurred in the MPLM group, the overall complication rates showed no significant difference between groups.

**Table 2.** Intraoperative and Post-Operative Data of MPLM and SPLM Groups

Intraoperative complications	Classification	MPLM (*n* = 77)	SPLM (*n* = 72)	*p* value^*^
Intraoperative blood loss (mL)^c^		50 (20, 75)	30 (20, 50)	0.095
Operative time (minutes)^a^		120.91 ± 49.18	99.85 ± 33.65	0.003
Intraoperative blood transfusion^b^		2 (2.6%)	0	0.497
Uterine cavity perforation^b^		1 (1.2%)	0	0.967
Conversion to laparotomy^b^		0	0	
Hemoglobin change (g/L)^a^		11.58 ± 8.25	11.42 ± 8.67	0.904
Postoperative complications^b^		7 (9.1%)	7 (9.7%)	0.895
	Gastrointestinal symptoms	1 (14.3%)	4 (57.1%)	0.266
	Respiratory symptoms	1 (14.3%)	1 (14.3%)	0.769
	Fever*	4 (57.1%)	0	0.070
	Postoperative blood transfusion*	2 (28.6%)	0	0.462
	Urinary symptoms	0	2 (28.6%)	0.462
	Leg DVT	1 (14.3%)	0	0.500
Time to first flatus (days)^a^		1.69 ± 0.47	1.40 ± 0.55	0.001
24-hour postoperative VAS^a^		2.70 ± 0.84	2.33 ± 0.65	0.004
Postoperative hospital stay (days)^a^		3.14 ± 1.07	2.99 ± 0.94	0.346

^a^Data are shown as the mean ± standard deviation.

^b^ *n* (%).

^c^ Median (interquartile range, IQR).

^*^ Among postoperative complications in the MPLM group, there were 4 patients with postoperative fever. Of these 4 patients, 2 had concurrent fever and postoperative blood transfusion, while the other 2 had isolated fever.

MPLM, multiport laparoscopic myomectomy; SPLM, single-port laparoscopic myomectomy; VAS, visual analog scale.

The postoperative hemoglobin decline was similar between the MPLM (11.58 ± 8.25 g/L) and SPLM (11.42 ± 8.67 g/L) groups (*p* > 0.05). The incidence of postoperative complications, including fever, gastrointestinal symptoms, and thrombosis, was not statistically significant. The SPLM group demonstrated significantly better outcomes in time to first flatus (1.40 ± 0.55 days) and VAS scores (2.33 ± 0.65) compared to the MPLM group (*p* < 0.05). No significant differences were observed between the groups in postoperative hospital stays.

### Subgroup analysis based on operative time

Table 3 shows that operative times were compared using different stratification criteria. For cases with either a single fibroid (118.00 ± 42.92 vs. 99.18 ± 34.90 minutes) or ≥4 fibroids (135.00 ± 40.44 vs. 92.22 ± 22.10 minutes), operative times were significantly longer in the MPLM group than in the SPLM group. For patients with multiple fibroids (2–3), neither group showed significant differences (*p* > 0.05) in operative time. For fibroids <8 cm in diameter, the SPLM group showed significantly shorter operative times (89.02 ± 26.38 vs. 109.87 ± 34.18 minutes, *p* < 0.05), while for fibroids ≥8 cm, the measured outcome showed insignificant differences between groups (*p* > 0.05). The comparison between FIGO types 2–5 (intramural fibroids) and type 6 (subserous fibroids) (Table 3) demonstrated that the SPLM group resulted in significantly shorter operative times for intramural fibroids (*p* < 0.05), whereas no significant difference was found for type 6 fibroids (*p* > 0.05).

**Table 3.** Subgroup Analysis Based on Operative Time (Minutes)

Influencing factors	Classification	MPLM	SPLM	*p* value
Number of fibroids^a^	1	118 ± 42.92	99.18 ± 34.90	0.030
	2	104.67 ± 31.48	96.92 ± 32.31	0.527
	3	138.57 ± 76.65	122.50 ± 39.59	0.636
	≥4	135.00 ± 40.44	92.22 ± 22.10	0.015
Fibroid diameter^a^	<8 cm	109.87 ± 34.18	89.02 ± 26.38	0.003
	≥8 cm	131.67 ± 58.81	117.59 ± 37.30	0.276
Fibroid type^a^	Type 5 and 2–5	125.26 ± 35.22	104.00 ± 31.78	0.016
	Type 6	121.79 ± 79.77	96.07 ± 31.52	0.141
Surgeon^a^	Surgeon A	118.50 ± 36.91	88.47 ± 25.86	0.000
	Surgeon A with single fibroid	118.75 ± 34.62	86.91 ± 27.47	0.003

^a^ Data are shown as the mean ± standard deviation.

MPLM, multiport laparoscopic myomectomy; SPLM, single-port laparoscopic myomectomy.

To control operator variability, we compared conventional multiport and single-port laparoscopic surgeries performed exclusively by Surgeon A, despite the study involving three experienced surgeons. Analysis revealed SPLM had significantly shorter operative time (88.47 ± 25.86 minutes vs. 118.50 ± 36.91 minutes, *p* < 0.05). For cases involving a single myoma, the SPLM group showed significant advantages over the MPLM in operative time (*p* < 0.05).

### Subgroup analysis based on blood loss

Table 4 compares intraoperative blood loss between the SPLM and MPLM groups across various stratification factors. Multiple myomectomy procedures showed comparable blood loss between the two groups. However, for cases with ≥4 fibroids, the SPLM group showed significantly less blood loss than the MPLM group (*p* < 0.05). For fibroids <8 cm in diameter, the SPLM group demonstrated lower median blood loss (20 mL, IQR 15–50) than the MPLM group (40 mL, IQR 20–50; *p* > 0.05), while no significant difference was found for fibroids ≥8 cm. Previous experience suggests, intramural fibroids (type 5 or 2–5) typically cause greater blood loss than subserosal fibroids (type 6). However, Table 4 demonstrates comparable outcomes between groups for both types. Notably, when performed by Surgeon A, the SPLM group yielded significantly reduced blood loss (median: 20 mL, IQR 20–50) compared to the MPLM group (median: 50 mL, IQR 30–162.5; *p* < 0.05).

**Table 4.** Subgroup Analysis Based on Blood Loss (mL)

Influencing factors	Classification	MPLM	SPLM	*p* value
Number of fibroids^a^	1	20 (20,50)	30 (20,50)	0.506
	2	30 (20,50)	20 (15,50)	0.555
	3	30 (20,75)	50 (20,250)	0.494
	≥4	100 (50,200)	20 (10,50)	0.004
Fibroid diameter^a^	<8 cm	40 (20,50)	20 (15,50)	0.052
	≥8 cm	50 (20,100)	50 (20,200)	0.655
Fibroid type^a^	Type 5 and 2–5	50 (20,100)	50 (20,100)	0.945
	Type 6	40 (20,62.5)	20 (20,50)	0.228
Surgeon^a^	Surgeon A	50 (30,162.5)	20 (20,50)	0.000
	Surgeon A with single fibroid	50 (35,100)	20 (20,50)	0.013

^a^ Data are shown as median (IQR).

IQR, interquartile range; MPLM, multiport laparoscopic myomectomy; SPLM, single-port laparoscopic myomectomy.

## Discussion

In our study, we found that single-port laparoscopy can successfully perform surgeries even when the number of fibroids exceeds four. The maximum number of treatable fibroids showed no significant difference between single-port and conventional multiport laparoscopy. For anterior wall fibroids and Type 6 or 7 fibroids, gynecologists prefer single-port laparoscopy to meet patients’ aesthetic preferences. However, conventional multiport laparoscopy remains preferred for complex cases. These include fibroids penetrating the entire uterine wall, large Type 2 submucosal fibroids, or cervical fibroids. Zhou et al.^11^ noted that SPLM requires appropriate conditions, and it should be used cautiously for patients with posterior wall fibroids, fibroids larger than 8 cm in diameter, or more than four fibroids. Kim et al.^7^ also found that SPLM is suitable for cases with fewer than five fibroids, with surgical outcomes and perioperative complications comparable to MPLM. However, some clinicians maintain that single-port laparoscopy is a more efficient and safer surgical approach, even suitable for patients with large or multiple fibroids, as it not only reduces trauma but also accelerates recovery.^12^

Our retrospective analysis found that the advantages of SPLM over MPLM primarily include surgical duration, postoperative gastrointestinal function recovery time, and postoperative pain. Both approaches showed comparable intraoperative blood loss and complication rates, consistent with published literature. Choi et al.^13^ found that SPLM required shorter operative time than conventional. Ranjan A et al.^9^ demonstrated that single-port laparoscopy provides superior postoperative outcomes, including reduced pain scores, improved scar satisfaction, and faster wound healing, establishing it as a viable alternative to conventional multiport laparoscopy. Compared to conventional multiport laparoscopy, single-port laparoscopy for uterine fibroid treatment did not show prolonged surgical duration, demonstrated less hemoglobin reduction, and yielded better cosmetic results. Thus, single-port laparoscopic surgery is superior to conventional multiport approaches in treating uterine fibroids.^14,15^

However, Zhou et al.^11^ found that SPLM required longer operative time. A meta-analysis showed no significant difference in operative time between single-port and conventional multiport laparoscopy. Single-port laparoscopy demonstrated superior cosmetic outcomes compared to conventional multiport laparoscopy, although no significant differences were found in conversion to laparotomy, postoperative bleeding, pain, bowel function recovery, or hospital stay.^16^ Another systematic review found that compared with conventional multiport laparoscopy, single-port laparoscopy prolonged operative duration in total hysterectomy and increased conversion rates to multiport or open surgery in myomectomy. Despite the cosmetic advantages of single-port laparoscopy, the authors could not conclude that it was superior to multiport approaches for benign gynecological diseases.^17^

During the first two decades of single-port laparoscopy development, most studies found that it required more time than conventional multiport laparoscopy.^18^ The limited operating space and instrument interference (known as the ‘chopstick effect’) compromised suturing efficiency, making clinicians cautious about choosing this approach. However, as surgeons gained proficiency and advanced equipment like articulating forceps, energy devices (for cutting/coagulation), and barbed sutures,^19^ single-port laparoscopy became more technically feasible. In this study, single-port laparoscopy demonstrated favorable outcomes for cases involving a single fibroid smaller than 8 cm. Even for challenging intramural fibroids such as Type 5, single-port laparoscopy showed time-saving benefits. When fibroids exceeded 8 cm, operative blood loss and duration showed no statistically significant difference from conventional multiport laparoscopy, attributed to the effective use of fibroid graspers and barbed sutures (specialized self-anchoring threads). During fibroid extraction, we minimized energy device usage to prevent excessive cauterization that could compromise healing. Although single-port laparoscopic surgery presents certain limitations in terms of operational convenience and comfort, the biases resulting from these constraints can be gradually mitigated through improved techniques and accumulated experience. Our analysis indicates that the overall operative time was shorter in the single-port laparoscopic group compared to the multiport approach. We attribute this reduction primarily to the more efficient specimen extraction facilitated by the single-port technique. Specifically, the use of a 3-cm single-port incision allows for more efficient retrieval of fibroid specimens compared to the extraction through multiple 1.5-cm incisions in multiport laparoscopy. However, our study did not systematically compare time allocation for specific surgical steps—such as fibroid enucleation, suturing of the uterine defect, specimen retrieval, and incision closure. Therefore, a stepwise time analysis across these distinct phases is warranted in future clinical investigations.

This study confirms the advantages of single-port laparoscopic procedures in treating benign gynecological diseases but requires rational and objective evaluation to prevent overuse. For high-bleeding-risk patients, multiport laparoscopy or laparotomy may be preferable. For uterus-preserving patients without fertility needs, retroperitoneal uterine artery ligation may achieve outcomes comparable to standard myomectomy.^19^ However, for gynecologists lacking thorough anatomical knowledge or with limited learning curves, retroperitoneal identification of the uterine artery may increase the risk of injury to adjacent structures like the ureter. Additionally, in the overall data analysis of this study, the proportion of anterior wall fibroids and subserosal fibroids (including types 6 and 7) was higher in the SPLM group compared to the MPLM group, whereas the proportion of fibroids in special locations and intramural fibroids was greater in the MPLM group. This discrepancy may stem from surgeons’ subjective preoperative assessment of surgical difficulty based on accumulated experience with different fibroid locations and types, leading to a preference for the perceived safer surgical approach. Within the confined pelvic space, most surgeons consider anterior or subserosal fibroids less challenging both during enucleation and suturing of the residual cavity compared to posterior wall fibroids or deeply embedded intramural fibroids. Unlike robotic surgery, which offers 360-degree articulation of instrument arms to overcome spatial limitations, conventional laparoscopic surgery lacks such flexibility. Therefore, the potential bias introduced by this selection tendency cannot be excluded in our retrospective analysis. Therefore, further randomized controlled clinical trials are needed to perform multidimensional comparisons between single-port and multiport laparoscopic surgery under specific influencing factors, in order to obtain high-quality and accurate research outcomes. Moreover, Special attention should focus on reproductive outcomes and complications (such as uterine rupture) in patients desiring pregnancy after laparoscopic surgery. In subsequent follow-ups, we will monitor patients’ reproductive outcomes to obtain more comprehensive clinical data.

## Conclusions

While minimally invasive techniques reduce trauma and enhance recovery, they demand greater surgical expertise. Single-port laparoscopy demonstrates significant clinical potential due to its advantages, but technique adoption must balance the surgeon’s experience and patient-specific factors. Despite proven safety and efficacy, single-port laparoscopy requires significant experience to master.

## Abbreviations Used

BMI: Body Mass Index
FDA: Food and Drug Administration
FIGO: International Federation of Gynecology and Obstetrics
IQR: InterQuartile Range
MPLM: MultiPort Laparoscopic Myomectomy
SPLM: Single-Port Laparoscopic Myomectomy
VAS: Visual Analog Scale
